# THE ROLE OF HETEROGENEITY IN NEUROCOGNITIVE AGING

**DOI:** 10.1093/geroni/igae098.1187

**Published:** 2024-12-31

**Authors:** Sylvain Moreno

**Affiliations:** Simon Fraser University, Vancouver, British Columbia, Canada

## Abstract

Neurocognitive aging research has predominantly adopted a linear approach, categorizing aging populations homogeneously. Researchers like Salthouse argue that this perspective perceives cognitive decline as almost linear from early adulthood. However, emerging findings challenge this view, highlighting significant variability in cognitive aging and proposing different subtyping methods to study the neurocognitive aging population. Our talk will propose a novel subtyping framework inspired by the Orchid and Dandelion theory from Boyce (2005). This new framework introduces a nuanced understanding of cognitive aging, categorizing individuals based on genetic and phenotypic characteristics to better capture the heterogeneity of cognitive abilities as people age. Using extensive datasets such as the UK Biobank and machine learning analytical techniques, our research investigates the impact of environmental factors on distinct cognitive aging subtypes. This approach diverges from the traditional linear models, revealing that lifestyle factors have varying impacts on different cognitive aging subtypes, underscoring the theory’s premise that some individuals (orchids) are more environmentally sensitive, affecting their cognitive aging process. In contrast, others (dandelions) show resilience. This paradigm shift towards recognizing cognitive aging’s inherent heterogeneity offers a more accurate and personalized understanding of aging, emphasizing the importance of subtyping in developing targeted interventions. Our findings advocate for a move beyond the “linear” methodology found in the literature, highlighting the need for a framework that accommodates the heterogeneity of cognitive aging.

